# The potential of Nutri-Score to differentiate foods in regulating food marketing to children

**DOI:** 10.1007/s00394-026-04041-4

**Published:** 2026-07-30

**Authors:** Anna Amberntsson, Mari Mohn Paulsen, Jorunn Sofie Randby, Kaja Lund-Iversen, Anne Lise Brantsæter, Lene Frost Andersen, Marianne Hope Abel

**Affiliations:** 1 Department of Food Safety, Norwegian Institute of Public Health, P.O. Box 222, Skoyen, 0213 Oslo, Norway; 2 Centre for Sustainable Diets, Norwegian Institute of Public Health, Oslo, Norway; 3 Department of Physical Health and Ageing, Norwegian Institute of Public Health, Oslo, Norway; 4 Department of Nutrition, Institute of Basic Medical Sciences, University of Oslo, Oslo, Norway; 5 Centre for Evaluation of Public Health Measures, Norwegian Institute of Public Health, Oslo, Norway

**Keywords:** Nutri-Score, Nutrient profiling, Child marketing restrictions, Comparative study

## Abstract

**Purpose:**

To evaluate the potential of Nutri-Score to identify foods unsuitable for marketing to children by assessing its agreement with two established nutrient profiling models: WHO-EURO 2023 and the Norwegian Regulation on Marketing (NORMA). Further, we explored the impact of additional criteria to improve agreement. Addition, we evaluated the agreement between a modified Nutri-Score, the «NewTools-score», and WHO-EURO 2023 and NORMA.

**Methods:**

Products from the Norwegian food composition table (N = 1,944) were used to explore scenarios for Nutri-Score (and NewTools-score) against WHO-EURO and NORMA. The scenarios were: 1) All products scoring C-E not permitted for marketing, 2) Same as scenario 1, but with additional criteria for trans fatty acids, non-sugar sweeteners (NSS), and added sugar for beverages. For NORMA, a third scenario was tested as an extension of scenario 2, additionally banning selected food categories from marketing. Agreement was assessed through cross classification.

**Results:**

Under scenario 1, 53% of products were classified as Nutri-Score C-E, and 47% and 23% not permitted for marketing under WHO-EURO and NORMA, respectively. Overall agreement between Nutri-Score C-E and WHO-EURO was 84%, and 66% with NORMA. However, Nutri-Score allowed some products within unhealthy food categories, e.g., ice creams with NSS. Applying additional criteria in scenarios 2 and 3 had important effects on banning such products. Results for the NewTools-score were almost identical.

**Conclusion:**

The Nutri-Score and the NewTools-score may be used for identifying a high proportion of products unsuitable for marketing to children. However, some additional criteria, including a complete ban from marketing in selected food categories, seems warranted to better protect children from exposure to marketing of unhealthy products.

**Supplementary Information:**

The online version contains supplementary material available at 10.1007/s00394-026-04041-4.

## Introduction

Unhealthy diets, overweight and obesity are among the leading causes of disability and death in Europe, and estimates suggest that they cause more than 1.2 million deaths across the continent each year [1].

Extensive research indicates that food marketing significantly influences the dietary preferences and consumption habits of both children and adults [2, 3]. Children and adolescents are exposed to marketing of unhealthy foods and beverages in various settings, such as stores, outdoor spaces, during sports, while watching television, and when using social media [4]. Several studies have shown that most marketing efforts particularly focus on promoting foods and beverages high in fat, salt, and/or added sugar, which contribute to poor diet quality [2, 5]. A strong association between the marketing of unhealthy foods and children’s dietary intake, along with the rising prevalence of childhood obesity, is well documented [6, 7]. As a consequence, the Member States of the World Health Organization (WHO) adopted in 2010 a set of recommendations on food marketing to children [8].

In food marketing regulations, nutrient profiling models serve the purpose to identify which foods to restrict from marketing towards children [8]. Nutrient profiling is defined by the WHO as “the science of classifying or ranking food according to their nutritional composition for reasons related to preventing disease and promoting health” [9]. One of the criteria suggested by the WHO is to develop nutrient profiling models for specific purposes [10]. In 2015, WHO Europe launched a nutrient profiling model specifically aimed for the purpose of restricting the marketing of unhealthy foods to children [11], which was updated in 2023 [12] (hereafter referred to as WHO-EURO). The WHO-EURO permits marketing of products within all food categories given that specified nutrient thresholds are met [12].

In October 2025, the Norwegian government adopted a marketing ban that prohibits the marketing of unhealthy foods particularly aimed at children and adolescents below the age of 18 [13]. The nutrient profiling model developed for the Norwegian regulation (hereafter referred to as the NORMA), is based on the recommendations from the WHO [14] and a national nutrient profiling model from 2013 when a self-regulatory scheme was implemented. One main structural difference between NORMA and WHO‑EURO is that WHO‑EURO applies nutrient thresholds across all food categories, whereas NORMA categorises foods into six categories that are always prohibited from being marketed to children regardless of nutritional content, five categories that are permitted only if specific nutrient criteria are met, and a remaining group of foods that are permitted without criteria [13].

The Nutri-Score is a five-colour front-of-pack (FOP) nutrition label that attempts to provide simplified information about the overall nutritional value of food products [15]. The nutrient profiling model behind the Nutri-Score was developed from a model originally developed by the Food Standards Agency in the UK as a tool to identify foods not permitted for television advertising to children [16]. The Nutri-Score was last updated in 2023 [17, 18] and may apply to all packaged foods that are under the EU food information to consumers regulation [19]. Hence, the Nutri-Score nutrient profiling model is developed for the purpose to guide consumers, and foods are ranked into five classes ranging from best nutritional value (dark green A) to lowest nutritional value (dark orange E). The Nutri-Score is used as a FOP-label in several European countries [17, 18].

In the research project ‘NewTools’ [20, 21], we have evaluated the Nutri-Score 2023 on its alignment with the Norwegian food-based dietary guidelines [22] and with a reference standard based on the ranking of foods by nutrition experts [23]. We have also assessed how food system actors view the performance of the Nutri-Score in the Norwegian food system [24]. Based on these findings [22–24], some important adjustments to the Nutri-Score underlying algorithm to improve alignment with the Nordic Nutrition Recommendations have been proposed [25], henceforth referred to as the «NewTools-score». The modifications targeted insufficient discrimination between whole grain and refined carbohydrate-rich foods; high-sugar breakfast cereals receiving favourable scores; the lack of scoring for fresh fruits and vegetables without nutrient declarations; limited recognition of the nutritional value of fish; and weak differentiation between full-fat and lean alternatives within cheeses, creams, and other fat-rich foods. To address these issues, seven revisions were introduced, primarily affecting the scoring of carbohydrate-rich foods, fat-rich foods, and fish products, improving the ability to capture variations in nutritional value. In total, 11% of food products in a Norwegian database of pre-packed foods obtained a different score with the NewTools-score compared to the Nutri-Score 2023 [25].

Across Europe, there is increasing interest in exploring whether nutrient profiling models can be harmonised and potentially serve multiple public health purposes. Recent EFSA scientific advice, requested by the European Commission, provides evidence to support the development of harmonised mandatory front‑of‑pack nutrition labelling and the establishment of nutrient profiles to underpin regulatory measures [26, 27]. Although EFSA’s work does not specifically address marketing to children, it highlights the need for profiling systems that can operate reliably across different regulatory contexts. At the same time, ongoing European initiatives such as the Joint Action PreventNCD (Task 5.3) aim to strengthen coordinated strategies on nutrition‑related risk factors, including work on food environment policies and front‑of‑pack labelling as part of wider NCD prevention efforts [27]. One subtask in the Joint Action project aims to design a harmonized nutrient profiling model that may be applied to all nutrition policies. At present, there are several nutrient profiling models available in Europe for different purposes and national contexts. It is therefore relevant to consider whether a nutrient profiling model developed for food labelling is suitable to differentiate foods in terms of nutritional value in the regulation of food marketing to children. Although the Nutri-Score was originally developed based on a nutrient model by the UK Food Standards Agency (the UK Ofcom NPM) designed to identify products that should not be marketed to children [29], the two differ in that the UK Ofcom NPM provides a dichotomous score, whereas the Nutri-Score ranks products across five levels.

In this study, we aimed to evaluate the potential of the Nutri-Score as a tool for defining food products unsuitable for marketing to children. The primary objectives were to assess the agreement of the Nutri-Score 2023 with two established nutrient profiling models specifically developed to define products for marketing restrictions: 1) the WHO-EURO 2023 and 2) the NORMA, and to explore the impact of additional criteria to the Nutri-Score on improving this agreement and to assess the nature of the disagreements identified. Additionally, we aimed to evaluate the agreement between a modified Nutri-Score version, the NewTools-score, and the same nutrient profiling models.

## Material and methods

### Data sources

The study included data from the Norwegian food composition table, version 2024 [29]. This version includes information about 2,049 food and beverage items (henceforth “foods”). Of the 2,049 foods, 94 were excluded as the products were not eligible for the nutrient profiling models investigated in the present study. These included alcoholic beverages (n = 21), supplements, herbs and spices (n = 27), products for special uses, baby foods, including breast-milk substitutes and commercially produced foods for infants and young children (n = 33), as well as home-cooked foods and dishes not representative of products sold in Norwegian stores (n = 13). We also excluded similar products if information was given in both the raw and ready-to-eat versions, e.g., for fruit squash (n = 6). In case of similar products, the ready-to-eat product was kept in the dataset. The final sample of foods used in the evaluation included 1,944 items.

### Nutrient profiling models

The WHO-EURO [12] and the NORMA [13] are considered as references in this study. Foods in the dataset in the present study were categorized according to the food categories in the WHO-EURO and coded as “permitted to be marketed” or “not permitted to be marketed”, and similarly according to the NORMA.

### WHO-EURO

The WHO-EURO (Table 1) was created for development of policies to restrict marketing of unhealthy foods to children aged 36 months and older [12]. Products are restricted from being marketed to children unless they meet specific nutritional criteria. It covers all food and non-alcoholic drinks divided into 22 food and beverage categories. In addition to the criteria listed in Table 1, the WHO-EURO prohibits marketing of all products containing more than 1 g/100 g of industrially produced trans fatty acids. However, no product in the Norwegian food composition table contains more than 1 g/100 g of industrially produced trans fatty acids.

**Table 1:** Criteria for foods permitted to be marketed according to the WHO nutrient profile model 2023 (WHO-EURO) [12] and the nutrient profiling model from the Norwegian Regulation prohibiting the marketing of certain foods particularly aimed at children (NORMA) [13], thresholds per 100 g

Product category	WHO-EURO 2023*	NORMA
Chocolate and sugar confectionery, energy bars, sweet toppings and desserts	Added sugar: 0 g Non-sugar sweeteners: 0 g	No products permitted
Cakes, sweet biscuits and pastries; other sweet bakery wares; and dry mixes for making such	Total fat: 3 g Added sugar: 0 g Non-sugar sweeteners: 0 g Sodium: 0.1 g	No products permitted
Savoury snacks	Added sugar: 0 g Non-sugar sweeteners: 0 g Sodium: 0.1 g	No products permitted
Beverages		
Juices	Total sugar: 0 g Non-sugar sweeteners: 0 g	Added sugar: 0 g Non-sugar sweeteners: 0 g
Dairy milk drinks	Total fat: 3 g Added sugar: 0 g Non-sugar sweeteners: 0 g	Added sugar: 0 g Non-sugar sweeteners: 0 g
Plant-based milks	Total fat: 3 g Added sugar: 0 g Non-sugar sweeteners: 0 g	Added sugar: 0 g Non-sugar sweeteners: 0 g
Energy drinks	Added sugar: 0 g Non-sugar sweeteners: 0 g	No products permitted
Soft drinks, bottled waters and other drinks	Added sugar: 0 g Non-sugar sweeteners: 0 g	No products permitted except for non-sweetened waters/mineral waters
Edible ices	Total fat: 3 g Added sugar: 0 g Non-sugar sweeteners: 0 g Sodium: 0.1 g	No products permitted
Breakfast cereals	Total fat: 17 g Total sugar: 12.5 g Sodium: 0.5 g	Total sugar: 12.5 g Fibre: 6 g
Yoghurt, sour milk, cream and similar foods	Total fat: 3 g Saturated fat: 1 g Total sugar: 12.5 g Sodium: 0.1 g	Total fat: 3 g Total sugar: 10 g Non-sugar sweeteners: 0 g Cream is permitted
Cheese	Total fat: 17 g Sodium: 0.5 g	All products permitted
Ready-made and convenience foods and composite dishes	Total fat: 17 g Saturated fat: 6 g Total sugar: 12.5 g Sodium: 0.5 g Energy: 225 kcal	Saturated fat: 4 g Salt: 1 g Energy: 225 kcal
Butter, other fats and oils	Saturated fat: 21 g Sodium: 0.5 g	All products permitted
Bread, bread products and crisp breads	Total fat: 17 g Total sugar: 12.5 g Sodium: 0.5 g	All products permitted
Fresh or dried pasta, rice and grains	Total fat: 17 g Total sugar: 12.5 g Sodium: 0.5 g	All products permitted
Fresh and frozen meat, poultry, fish and similar	Total fat: 17 g	All products permitted
Processed meat, poultry, fish and similar	Total fat: 17 g Sodium: 0.5 g	All products permitted
Fresh and frozen fruit, vegetables and legumes	All products permitted	All products permitted
Processed fruit and vegetables	Total fat: 17 g Total sugar: 12.5 g Added sugar: 0 g Sodium: 0.5 g	All products permitted
Savoury plant-based foods/ meat analogues	Total fat: 17 g Added sugar: 0 g Non-sugar sweeteners: 0 g Sodium: 0.5 g	All products permitted
Sauces, dips and dressings	Total fat: 17 g Added sugar: 0 g Non-sugar sweeteners: 0 g Sodium: 0.5 g	All products permitted

NSS: Non-sugar sweeteners. *In addition to the criteria listed in the table, the WHO‑EURO model prohibits marketing of any product containing more than 1 g/100 g of industrially produced trans fatty acids. No products in the Norwegian food composition table exceeded this threshold; therefore, this criterion did not affect the classification in the present study

### NORMA

The NORMA (Table 1) is a nutrient profiling model comprising 11 specific food categories [12]. First, the NORMA specifies six food categories that cannot be marketed towards children, regardless of nutritional content: 1) Chocolate and confectionary, energy bars, sweet spreads, and desserts; 2) Cakes, biscuits, and other sweet and/or fatty pastries; 3) Snacks; 4) Ice cream; 5) Energy drinks; and 6) Soft drinks, cordials, and similar beverages [13]. The rationale for this restriction is to preclude the promotion of food categories that are dominated by foods high in fat, salt, and/or added sugar, while providing few beneficial nutrients. Additionally, products within the following five categories (categories 7–11) are restricted from being marketed to children unless they meet specific nutritional criteria (Table 1): 7) Juice and similar products; 8) Milk and plant-based drinks; 9) Breakfast cereals; 10) Yoghurt and similar products; 11) Ready meals and composite dishes. Some products within categories 7–11 are recommended in the national food-based dietary guidelines [30], while others should be consumed in moderation due to potentially high levels of fat, salt and/or added sugar. Foods not covered by categories 1–11 are according to the NORMA permitted to be marketed to children, regardless of nutritional content. In the present study, for simplification, we define these foods as category 12, although they belong to a wide variety of food categories.

### Calculation of the Nutri-Score and the NewTools-score

We calculated scores for all food products in the dataset, including whole foods (e.g., vegetables and fresh fish), using the three Nutri-Score 2023 algorithms: general foods [31], fats, oils, nuts, and seeds [31], and beverages [32]. Energy, total sugar, saturated fat, salt, non-sugar sweeteners (in beverages), protein, fibre and the proportion of fruit, vegetables, and legumes were used to calculate the Nutri-Score. The food composition table lacked information about the content of fruit, vegetables, and legumes. To estimate the fruit, vegetable and legume proportion, the component was set to 100% for all pure fruit, vegetables and legumes, as defined by Nutri-Score [33]. For composite foods, information from similar or the actual products was used to estimate the proportion. For beverages, we determined the presence or absence of non-sugar sweeteners manually using product descriptions in the food composition table, online ingredient lists, or comparable products.

Likewise, we used the same variables plus a variable for the content of fish and total carbohydrates for calculation of the NewTools-score [25].

### Scenarios for testing the potential of Nutri-Score and NewTools-score in food marketing restrictions

While the WHO-EURO and the NORMA classify foods dichotomously as permitted or not permitted to be marketed to children, the Nutri-Score and NewTools-score classifies food into five classes (A-E). To facilitate investigation of the agreement between the Nutri-Score/NewTools-score and the WHO-EURO and the NORMA, binary scenarios of the Nutri-Score/NewTools-score were created to define foods as permitted or not permitted to be marketed to children.

First, the distribution of the Nutri-Score (A-E) with the WHO-EURO and the NORMA was explored through cross-classification to identify the cutoff for Nutri-Score providing the highest agreement. The scenarios had increasing levels of complexity to investigate the feasibility of applying additional criteria to the Nutri-Score/NewTools-score to enhance agreement with the WHO-EURO and the NORMA. The scenarios were defined as follows:

**Nutri-Score**/NewTools-score** scenario 1)** Food products are not permitted if they have a Nutri-Score/NewTools-score of C-E. This cut-off was selected as it showed best overlap with the WHO-EURO and the NORMA, and because products scoring A or B are generally considered nutritionally favourable, corresponding to the “green” Nutri‑Score classes. Best overlap was defined as the highest percentage agreement between the Nutri-Score/NewTools-score and the WHO-EURO and the NORMA, depending on where the cut-off in Nutri-Score class was placed.

**Nutri-Score**/NewTools-score** scenario 2)** Same as scenario 1, but with the additional criteria that solid foods containing ≥ 1 g of industrially produced trans fatty acids, solid foods and beverages containing non-sugar sweeteners (NSS), or beverages with added sugar, are not permitted. These additional criteria align with the strict regulations on NSS and added sugar in both the WHO-EURO [12] and the NORMA [13].

Since the NORMA defines specific food categories where either all or none of the products are permitted to be marketed, a third scenario was created to incorporate these criteria. Agreement with this third scenario was assessed against the NORMA only. The third scenario was defined as:

**Nutri-Score**/NewTools-score** scenario 3)** Same as scenario 2, and additionally, no products in NORMA categories 1–6 are permitted to be marketed. For categories 7–11, the criteria from scenario 2 apply. All products in category 12 (other foods) are permitted.

### Data analysis

Cross-classification was used to assess the agreement between the Nutri-Score for each of the three scenarios described above, separately for the WHO-EURO and the NORMA. Agreement was described as the proportion (%) of foods that met the following conditions:

1. Not permitted to be marketed according to both the Nutri-Score scenario and i) the WHO-EURO or ii) the NORMA.
2. Permitted to be marketed according to both the Nutri-Score scenario and i) the WHO-EURO or ii) the NORMA.

In addition, disagreement was described as the proportion (%) of foods that met the following conditions:

1. Permitted to be marketed according to the Nutri-Score scenario but not permitted according to i) the WHO-EURO or ii) the NORMA.
2. Not permitted to be marketed according to the Nutri-Score scenario but permitted by i) the WHO-EURO or ii) the NORMA.

Similar analyses were done for the NewTools score, and detailed results are included in the Supplementary Material.

Statistical analyses were conducted in Stata/SE 17.0.

### Ethics

Ethical approval was not required as no human or animal subjects were involved in this study.

## Results

Among our sample of 1,944 products in the Norwegian food composition table, 919 products (47%) were classified as not permitted to be marketed according to the WHO-EURO, and 455 products (23%) according to the NORMA. In total, 1,037 products (53%) were classified as Nutri-Score C-E.

There was a large overlap in the scoring of nutritional value using the Nutri-Score and the NewTools-score, with 91% of products obtaining the same score (A-E) and 98.3% obtaining the same classification of A/B vs. C-E (Supplementary Table 1). Due to the large overlap, all results for the NewTools-score are presented in the Supplementary Materials.

### Comparing the Nutri-Score and the WHO-EURO

Table 2 presents the distribution of the Nutri-Score for foods in the Norwegian food composition table within each food category defined by the WHO-EURO. In the first three categories, which comprise confectionery, sweet bakery wares, and savoury snacks, 91% of the products were not permitted to be marketed according to the criteria from the WHO-EURO. Among these, 5% received Nutri-Score A or B. No juices or energy drinks in the dataset were permitted to be marketed by the WHO-EURO, while 10% of all juices had Nutri-Score B (none had an A). Approximately half of the dairy and plant-based milks, as well as products in the category “soft drinks, bottled waters, and other drinks”, were not permitted by the WHO-EURO, of which 16% had Nutri-Score A or B. Only water can get Nutri-Score A in the beverage category.

**Table 2:** Foods and beverages in the Norwegian food composition table (N = 1,944) within each category of the WHO nutrient profile model 2023 (the WHO-EURO), splits of not permitted and permitted products, and Nutri-Score distribution

WHO category number	Food category	Categorization by WHO-EURO	Nutri-Score N (%)^1^				
			A	B	C	D	E
	All foods	Not permitted (n = 919)	40 (4)	48 (5)	213 (23)	339 (37)	279 (30)
		Permitted (n = 1,025)	653 (64)	166 (15)	170 (17)	29 (3)	7 (1)
1	Chocolate and sugar confectionery, energy bars, sweet toppings and desserts	Not permitted (n = 87)	2 (2)	2 (2)	17 (20)	28 (32)	38 (44)
		Permitted (n = 7)	5 (71)	0 (0)	0 (0)	0 (0)	2 (29)
2	Cakes, sweet biscuits and pastries; other sweet bakery wares	Not permitted (n = 179)	2 (1)	4 (2)	29 (16)	52 (29)	92 (51)
		Permitted (n = 0)	0 (0)	0 (0)	0 (0)	0 (0)	0 (0)
3	Savoury snacks	Not permitted (n = 36)	0 (0)	5 (14)	13 (36)	12 (33)	6 (17)
		Permitted (n = 23)	18 (78)	4 (17)	1 (4)	0 (0)	0 (0)
4.1	Juices	Not permitted (n = 30)	0 (0)	3 (10)	12 (40)	10 (33)	5 (17)
		Permitted (n = 0)	0 (0)	0 (0)	0 (0)	0 (0)	0 (0)
4.2	Dairy milk drinks	Not permitted (n = 28)	0 (0)	3 (11)	11 (39)	3 (11)	11 (39)
		Permitted (n = 28)	0 (0)	25 (89)	3 (11)	0 (0)	0 (0)
4.3	Plant based milk	Not permitted (n = 12)	0 (0)	7 (58)	1 (8)	0 (0)	4 (3)
		Permitted (n = 15)	0 (0)	2 (13)	7 (47)	6 (40)	0 (0)
4.4	Energy drinks	Not permitted (n = 2)	0 (0)	0 (0)	1 (50)	0 (0)	1 (50)
		Permitted (n = 0)	0 (0)	0 (0)	0 (0)	0 (0)	0 (0)
4.5	Soft drinks, bottled waters and other drinks	Not permitted (n = 22)	0 (0)	0 (0)	5 (23)	3 (14)	14 (64)
		Permitted (n = 16)	7 (44)	6 (38)	2 (12)	1 (6)	0 (0)
5	Edible ices	Not permitted (n = 30)	3 (10)	0 (0)	6 (20)	17 (57)	4 (13)
		Permitted (n = 0)	0 (0)	0 (0)	0 (0)	0 (0)	0 (0)
6	Breakfast cereals	Not permitted (n = 13)	1 (8)	2 (15)	6 (46)	4 (31)	0 (0)
		Permitted (n = 12)	5 (42)	1 (8)	6 (50)	0 (0)	0 (0)
7	Yoghurt, sour milk, cream and similar foods	Not permitted (n = 33)	2 (6)	8 (24)	13 (39)	10 (30)	5 (15)
		Permitted (n = 15)	6 (40)	4 (27)	4 (27)	1 (6)	0 (0)
8	Cheese	Not permitted (n = 56)	0 (0)	0 (0)	5 (9)	37 (66)	14 (25)
		Permitted (n = 13)	1 (8)	0 (0)	4 (31)	3 (23)	5 (38)
9	Ready-made and convenience foods and composite dishes	Not permitted (n = 48)	0 (0)	1 (2)	25 (52)	20 (42)	2 (4)
		Permitted (n = 102)	24 (24)	31 (30)	42 (41)	5 (5)	0 (0)
10	Butter, other fats and oils	Not permitted (n = 21)	0 (0)	0 (0)	0 (0)	5 (24)	16 (76)
		Permitted (n = 31)	0 (0)	10 (32)	17 (55)	4 (13)	0 (0)
11	Bread, bread products and crisp breads	Not permitted (n = 20)	1 (5)	0 (0)	7 (35)	8 (40)	4 (20)
		Permitted (n = 90)	42 (47)	20 (22)	28 (31)	0 (0)	0 (0)
12	Fresh or dried pasta, rice and grains	Not permitted (n = 3)	0 (0)	0 (0)	1 (33)	2 (67)	0 (0)
		Permitted (n = 103)	65 (63)	27 (26)	11 (11)	0 (0)	0 (0)
13	Fresh and frozen meat, poultry, fish and similar	Not permitted (n = 12)	0 (0)	2 (17)	1 (8)	8 (67)	1 (8)
		Permitted (n = 126)	108 (86)	5 (4)	11 (9)	2 (2)	0 (0)
14	Processed meat, poultry, fish and similar	Not permitted (n = 165)	4 (2)	5 (3)	21 (13)	87 (53)	48 (29)
		Permitted (n = 159)	105 (66)	19 (12)	29 (18)	6 (4)	0 (0)
15	Fresh and frozen fruit, vegetables and legumes	Not permitted (n = 0)	0 (0)	0 (0)	0 (0)	0 (0)	0 (0)
		Permitted (n = 215)	210 (98)	3 (1)	1 (0.5)	1 (0. 5)	0 (0)
16	Processed fruit and vegetables	Not permitted (n = 59)	15 (25)	3 (5)	25 (42)	14 (24)	2 (3)
		Permitted (n = 51)	45 (88)	6 (12)	0 (0)	0 (0)	0 (0)
17	Savoury plant-based foods/meat analogues	Not permitted (n = 28)	7 (25)	2 (7)	7 (25)	6 (21)	6 (21)
		Permitted (n = 11)	9 (82)	1 (9)	1 (9)	0 (0)	0 (0)
18	Sauces, dips and dressings	Not permitted (n = 35)	3 (9)	1 (3)	7 (20)	13 (37)	11 (31)
		Permitted (n = 8)	3 (38)	2 (25)	3 (38)	0 (0)	0 (0)

^1^Percentages may not sum to 100% due to rounding

Notably, although three products in category 5 (‘Edible ices’) received Nutri-Score A, 100% were classified as not permitted under the WHO-EURO. Across the remaining 13 categories, 34% of products fell into the not permitted group according to WHO-Euro, and among these, 88% attained Nutri-Score C-D. 

### Evaluating agreement in food marketing permissions between the WHO-EURO and different scenarios of the Nutri-Score

**Fig. 1:**
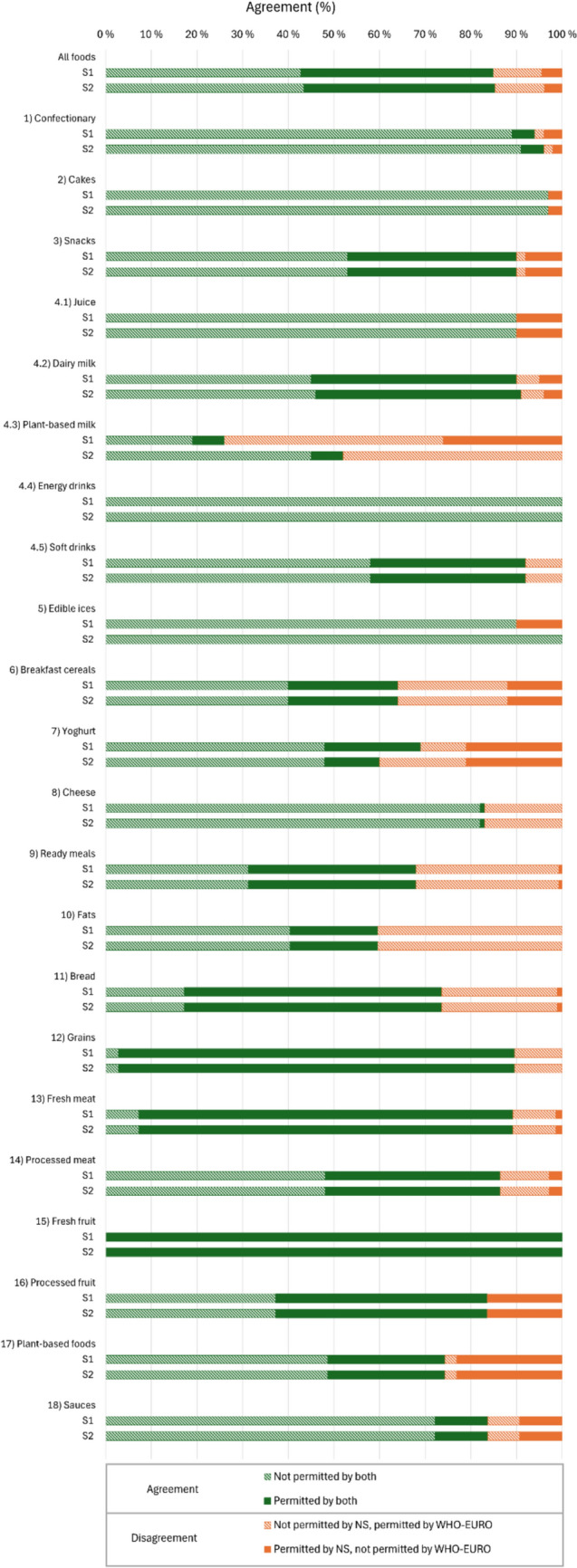
Overall and within food category agreement between the WHO-EURO and the two scenarios of how the Nutri-Score could be applied to regulate which food items could be permitted to be marketed to children, in the Norwegian food composition table (N = 1,944). Nutri-Score scenario 1 (S1) – Products are not permitted for marketing if they have Nutri-Score C-E. Nutri-Score scenario 2 (S2) – Products are not permitted for marketing if they have Nutri-Score C-E, contain ≥ 1 g of industrially produced trans fatty acids in solid foods, include non-sugar sweeteners in solid foods and beverages, or have added sugar in beverages. Abbreviations: NS, Nutri-Score; WHO-EURO, the WHO nutrient profile model 2023

The overall agreement in food marketing permissions between Nutri-Score scenario 1 and the WHO-EURO was 84%, and 85% between scenario 2 and the WHO-EURO (Fig. 1).

As regards disagreements in **scenario 1**, the products classified as not permitted to be marketed according to the WHO-EURO, but permitted according to the Nutri-Score, accounted for 4.5% (n = 88, full orange in Fig. 1). This included jam with NSS, pancakes and waffles, salted nuts, juice, dairy or plant-based milk, sugar-free ice cream with NSS, breakfast cereals, plain yoghurt, fatty fish, canned fruit, and plant-based meat analogues. In **scenario 2**, 3.9% of the products (n = 75, full orange in Fig. 1) were still in disagreement. Table 3 provides examples of products in disagreement from each of the food categories from both scenario 1 and 2.

**Table 3:** Number and examples of products in disagreement in scenario 1^1^ and 2^2^, being permitted by the Nutri-Score but not by the WHO nutrient profile model 2023 (WHO-EURO)

WHO category	Scenario 1^1^ N in disagreement (product examples)	Scenario 2^1^ N in disagreement (product examples)
1	N = 4 (Fruit soup, from powder, sugar free jam with NSS, sugar free pastilles with NSS)	N = 2 (Fruit soup, from powder)
2	N = 6 (Pancakes, waffles, crackers, custard)	N = 6 (Pancakes, waffles, crackers, custard)
3	N = 5 (Salted nuts)	N = 5 (Salted nuts)
4.1	N = 3 (Juices (tomato, cucumber/spinach, lemon), no added sugar)	N = 3 (Juices (tomato, cucumber/spinach, lemon), no added sugar)
4.2	N = 3 (Whole milk, 3.5% fat, < 3% added sugar)	N = 2 (Whole milk, 3.5% fat, < 3% added sugar)
4.3	N = 7 (Plant-based milk, < 3% added sugar)	N = 0
5	N = 3 (Sugar free ice cream with NSS)	N = 0
6	N = 3 (Breakfast cereals, fibre > 9%, sugar 13–17%)	N = 3 (Breakfast cereals, fibre > 9%, sugar 13–17%)
7	N = 10 (Yoghurt, plain, 3.4% fat)	N = 10 (Yoghurt, plain, 3.4% fat)
9	N = 1 (Tortellini)	N = 1 (Tortellini)
11	N = 1 (Wheat germ)	N = 1 (Wheat germ)
13	N = 2 (Raw mackerel)	N = 2 (Raw mackerel)
14	N = 9 (Pan fried salmon)	N = 9 (Pan fried salmon)
16	N = 18 (Lentils and beans salad, canned fruit in syrup)	N = 18 (Lentils and beans salad, canned fruit in syrup)
17	N = 9 (Falafel, soy burger)	N = 9 (Falafel, soy burger)
18	N = 4 (Stew base)	N = 4 (Stew base)

NSS, non-sugar sweeteners

^1^Nutri-Score C-E not permitted for marketing

^2^Same as scenario 1, but with additional criteria for trans fatty acids, non-sugar sweeteners, and added sugar for beverages

In **scenario 1,** the products that were classified differently by the WHO‑EURO and the Nutri‑Score (permitted to be marketed by the WHO-EURO, but not by the Nutri-Score), represented 11% (n = 206, striped orange in Fig. 1). This included products from all food categories, for example honey, dried fruits and nuts mixes, plant-based milks, breakfast cereals, sour milk, cheese, ready meals, margarine, and refined bread, rice and pasta. In **scenario 2**, the same products were classified differently as in scenario 1 (11%, n = 210, striped orange in Fig. 1). Four sour milk products that either contained NSS or had added sugar < 1.5 g were also included, as the WHO-EURO does not include criteria for NSS or added sugar within this food category.

Results for the agreement between the NewTools-score and the WHO-EURO were almost identical to those for the Nutri-Score (Supplementary Fig. 1). 

### Comparing the Nutri-Score and the NORMA

Table 4 presents the distribution of the Nutri-Score for foods in the Norwegian food composition table within each food category defined by the NORMA. Among the products in the first six categories where no products are permitted for marketing by the NORMA, 5% received Nutri-Score A or B. Notably, three products in the category ‘Chocolate and confectionery, energy bars, sweet spreads’ and three in the ‘Ice cream’ category achieved Nutri-Score A, including jams with NSS, sugar free pastilles with NSS, and ice creams with NSS and added fibre. In the remaining five food categories covered by the NORMA (category 7–11, n = 276), 43% were not permitted to be marketed, of which 82% had Nutri-Score C-E.

**Table 4:** Foods and beverages in the Norwegian food composition table (N = 1,944) within each category of the nutrient profiling model from the Norwegian Regulation prohibiting the marketing of certain foods particularly aimed at children (NORMA), splits of not permitted and permitted products, and Nutri-Score distribution

NORMA category number	Food category	Categorization by NORMA	Nutri-Score N (%)^1^				
			**A**	**B**	**C**	**D**	**E**
	All foods	Not permitted (n = 452)	11 (2)	28 (6)	112 (25)	129 (28)	175 (39)
		Permitted (n = 1,492)	682 (46)	186 (13)	271 (18)	239 (16)	111 (7)
1	Chocolate and confectionary, energy bars, sweet spreads, and desserts	Not permitted (n = 116)	3 (3)	4 (4)	19 (17)	33 (30)	52 (47)
		Permitted (n = 0)	0 (0)	0 (0)	0 (0)	0 (0)	0 (0)
2	Cakes, biscuits, and other sweet and/or fatty pastries	Not permitted (n = 140)	0 (0)	3 (2)	12 (9)	40 (29)	84 (60)
		Permitted (n = 0)	0 (0)	0 (0)	0 (0)	0 (0)	0 (0)
3	Snacks	Not permitted (n = 34)	0 (0)	5 (15)	12 (35)	13 (38)	4 (12)
		Permitted (n = 0)	0 (0)	0 (0)	0 (0)	0 (0)	0 (0)
4	Ice cream	Not permitted (n = 30)	3 (10)	0 (0)	6 (20)	17 (57)	4 (13)
		Permitted (n = 0)	0 (0)	0 (0)	0 (0)	0 (0)	0 (0)
5	Energy drinks	Not permitted (n = 2)	0 (0)	0 (0)	1 (50)	0 (0)	1 (50)
		Permitted (n = 0)	0 (0)	0 (0)	0 (0)	0 (0)	0 (0)
6	Soft drinks, cordials, and similar drinks	Not permitted (n = 21)	0 (0)	0 (0)	6 (29)	2 (10)	13 (62)
		Permitted (n = 0)	0 (0)	0 (0)	0 (0)	0 (0)	0 (0)
7	Juice and similar products	Not permitted (n = 3)	0 (0)	0 (0)	2 (67)	0 (0)	1 (33)
		Permitted (n = 27)	0 (0)	2 (7)	10 (37)	10 (37)	5 (19)
8	Milk and plant-based drinks	Not permitted (n = 35)	0 (0)	9 (26)	11 (31)	3 (9)	12 (34)
		Permitted (n = 55)	0 (0)	33 (60)	16 (29)	6 (11)	0 (0)
9	Breakfast cereals	Not permitted (n = 19)	2 (11)	3 (16)	10 (53)	4 (21)	0 (0)
		Permitted (n = 9)	6 (67)	1 (11)	2 (22)	0 (0)	0 (0)
10	Yoghurt and similar products	Not permitted (n = 12)	3 (25)	3 (25)	6 (50)	0 (0)	0 (0)
		Permitted (n = 6)	3 (50)	2 (33)	1 (17)	0 (0)	0 (0)
11	Ready meals and composite dishes	Not permitted (n = 49)	0 (0)	1 (2)	27 (55)	17 (35)	4 (8)
		Permitted (n = 61)	18 (30)	21 (34)	22 (36)	0 (0)	0 (0)
12	Other foods	All permitted (n = 1,331)	655 (49)	127 (10)	221 (17)	222 (17)	106 (8)

^1^Percentages may not sum to 100% due to rounding

### Evaluating agreement in food marketing permissions between the NORMA and different scenarios of the Nutri-Score

In **scenario 1**, there was an overall agreement of 66%. In **scenario 2**, the overall agreement was 67%. In **scenario 3**, and for category 12, the overall agreement was 96%.

Within category 1–11 (products covered by the NORMA), the overall agreement for Nutri-Score scenario 1 and 2 was 82% and 85%, respectively.

**Fig. 2:**
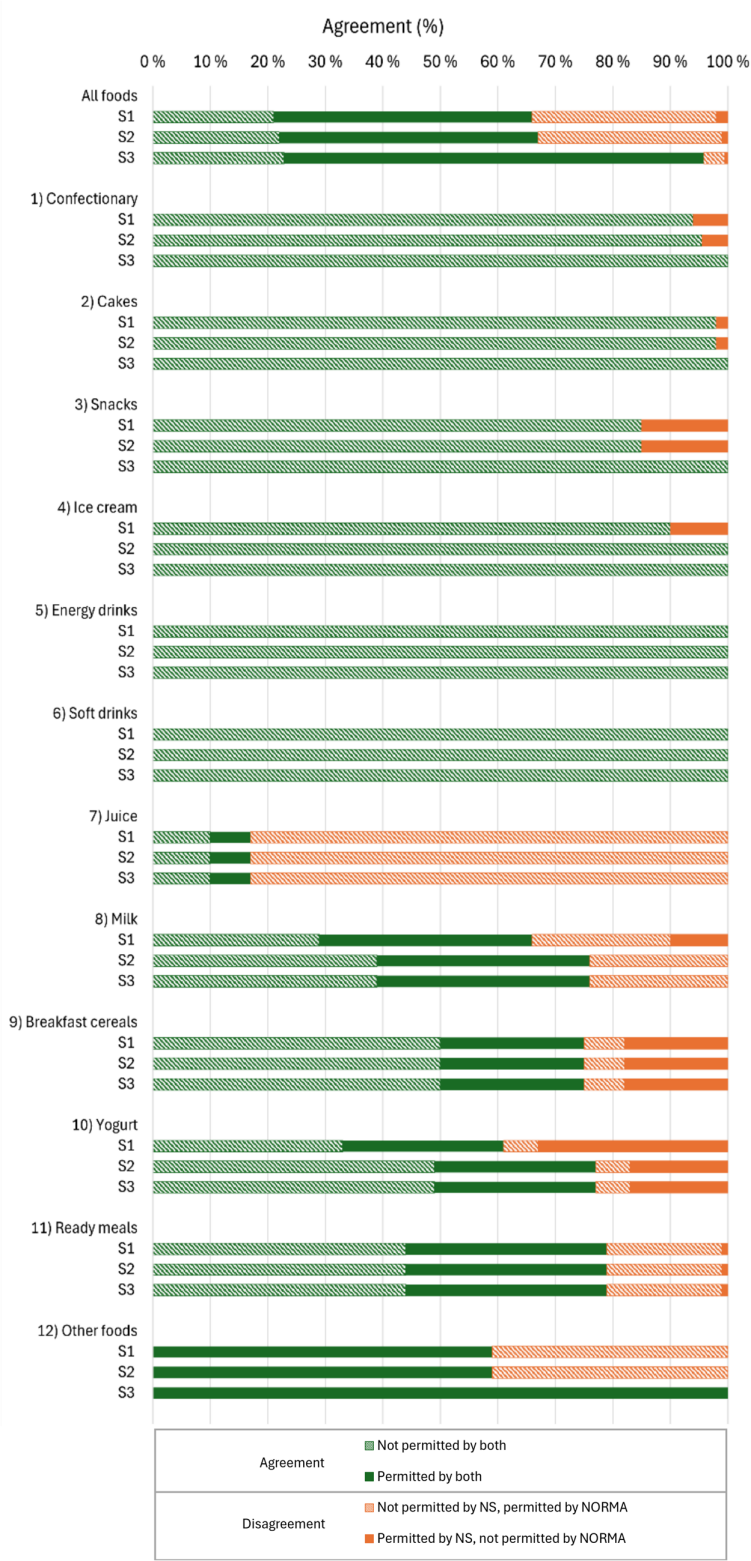
Overall and within food category agreement between the NORMA and the three scenarios of how the Nutri-Score could be applied to regulate which food items could be permitted to be marketed to children, in the Norwegian Food Composition Table (N = 1,944). Nutri-Score scenario 1 (S1) – Products are not permitted for marketing if they have Nutri-Score C-E. Nutri-Score scenario 2 (S2) – Products are not permitted for marketing if they have Nutri-Score C-E, contain ≥ 1 g of industrially produced trans fatty acids in solid foods, include non-sugar sweeteners in solid foods and beverages, or have added sugar in beverages. Nutri-Score scenario 3 (S3) – Products are not permitted for marketing if they have Nutri-Score C-E, contain ≥ 1 g of industrially produced trans fatty acids in solid foods, include non-sugar sweeteners in solid foods and beverages, or have added sugar in beverages. In addition, no products in category 1-6 were permitted to be marketed, but all products in category 12 were. Abbreviations: NS, Nutri-Score; NORMA, the nutrient profiling model from the Norwegian Regulation prohibiting the marketing of certain foods particularly aimed at children

In **scenario 1**, products not permitted according to the NORMA, but permitted according to the Nutri-Score, represented 2.0% (n = 39, full orange in Fig. 2). These included jam with NSS, pancakes and waffles, salted nuts, dairy or plant-based milk, sugar free ice cream with NSS, plain yoghurt, breakfast cereals, and a pre-packed salad. Table 5 provides examples of products from each of the food categories with disagreements from the three different scenarios. In **scenario 2**, only 21 products (1.1%) disagreed (not permitted according to the NORMA, but permitted according to the Nutri-Score, full orange in Fig. 2). In **scenario 3**, only nine products (0.5%) remained in disagreement (not permitted according to the NORMA but permitted according to the Nutri-Score, full orange in Fig. 2). These included plain yoghurts, breakfast cereals, and a pre-packed salad (Table 5).

**Table 5:** Number and examples of products in disagreement in scenario 1^1^, 2^2^ and 3^3^, being permitted by the Nutri-Score but not by the nutrient profiling model from the Norwegian Regulation prohibiting the marketing of certain foods particularly aimed at children (NORMA)

NORMA category	Scenario 1^1^ N in disagreement (product examples)	Scenario 2^2^ N in disagreement (product examples)	Scenario 3^3^ N in disagreement (product examples)
1	N = 7 (Fruit soup, from powder, custard, from powder, sugar free jam with NSS, sugar free pastilles with NSS)	N = 4 (Fruit soup, from powder, custard, from powder, sugar free jam with NSS, sugar free pastilles with NSS)	N = 0
2	N = 3 (Pancakes, waffles, crackers)	N = 3 (Pancakes, waffles, crackers)	N = 0
3	N = 5 (Salted nuts)	N = 5 (Salted nuts)	N = 0
4	N = 3 (Sugar free ice cream with NSS)	N = 0	N = 0
8	N = 9 (Plant-based milk, < 3% added sugar, flavoured dairy milk, < 3% added sugar)	N = 0	N = 0
9	N = 5 (Breakfast cereals/muesli, fibre > 9%, sugar 13–17%, puffed rice, non-sweetened)	N = 5 (Breakfast cereals/muesli, fibre > 9%, sugar 13–17%, puffed rice, non-sweetened)	N = 5 (Breakfast cereals/muesli, fibre > 9%, sugar 13–17%, puffed rice, non-sweetened)
10	N = 6 (Yoghurt with NSS, yoghurt, plain, 3.4% fat)	N = 3 (Yoghurt with NSS, yoghurt, plain, 3.4% fat)	N = 3 (Yoghurt with NSS, yoghurt, plain, 3.4% fat)
11	N = 1 (Caesar salad)	N = 1 (Caesar salad)	N = 1 (Caesar salad)

NSS, non-sugar sweeteners

^1^Nutri-Score C-E not permitted for marketing

^2^Same as scenario 1, but with additional criteria for trans fatty acids, non-sugar sweeteners, and added sugar for beverages

^3^No products in NORMA categories 1–6 are permitted to be marketed. For categories 7–11, the criteria from scenario 2 apply. All products in category 12 (other foods) are permitted

Within category 1–11 (n = 613), the same products (n = 39 and n = 21) disagreed (not permitted according to the NORMA) for Nutri-Score scenario 1 and 2 respectively, as described above and in Table 5.

In **scenarios 1** and** 2**, products permitted according to the NORMA, but not permitted according to the Nutri-Score, represented 32% (n = 621, striped orange in Fig. 2). Of these products, more than 88% (n = 549) were not covered by the NORMA (category 12, “other foods”), including foods such as cheese, bread, processed meat, and sauces. Additionally, this category included juices and smoothies (7–12 g sugar/100 g), dairy milk (> 3.5% fat) and plant-based milk, ready meals, breakfast cereals, and yoghurt. In **scenario 3**, 3.5% (n = 72, striped orange in Fig. 2) remained in disagreement, consisting of juices and smoothies, dairy milk and plant-based milk.

Results for the agreement between the NewTools-score and the NORMA were almost identical to those for the Nutri-Score (Supplementary Fig. 2).

## Discussion

This study evaluated the potential of the Nutri-Score and the NewTools-score to define food products unsuitable for marketing to children, by assessing their agreement with the WHO-EURO and the NORMA. Our results showed high overall agreement (84%) when comparing restricting Nutri-Score C-E with the WHO-EURO. Similarly, moderately high agreement (66%) was found between restricting Nutri-Score C-E and the NORMA (82% within the food categories covered by the NORMA). Despite high agreement, if used without additional criteria, the Nutri-Score would allow some products within unhealthy food categories to be marketed to children, such as sugar free pastilles, ice creams and yoghurts with NSS. Additional criteria to Nutri-Score, concerning trans fatty acids, NSS, and added sugar in beverages, had important effects on banning such products in our dataset. Although the NewTools-score differs from the Nutri-Score in certain aspects, we found that the performance of the two scores was almost identical in this setting. 

### Products in disagreement and importance of additional criteria

To consider the potential of the Nutri-Score to define food products for the purpose of marketing restrictions towards children, of particular interest in this study were the products not permitted by the WHO-EURO or the NORMA but permitted by the Nutri-Score (e.g. obtaining A or B). As presented in the results, across all scenarios, 3.9–4.5% of all products were not permitted by the WHO-EURO, but permitted by the Nutri-Score, and similarly 0.5–2.0% of products were not permitted by the NORMA but permitted by the Nutri-Score. When using only the Nutri-Score C-E criteria, some products scoring A or B with poor nutritional value were permitted to be marketed, such as canned or pickled fruit and vegetables, pancakes and waffles, sugar-free ice cream with NSS, and breakfast cereals with high sugar content. Adding three simple additional criteria to the Nutri-Score on no trans fatty acids, no NSS and no added sugar in beverages banned most of these products from marketing. This may, however, be influenced by the selection of products in the dataset. Incorporating additional criteria, as suggested in the present study, is thus important if the Nutri-Score should be used as a nutrient profiling model to regulate food marketing towards children. It is also worth noting that most products in disagreement between Nutri-Score and the WHO-EURO were foods with moderate to high nutritional value, such as nuts, juice, plain yoghurt, dairy or plant-based milk, plant-based meat analogues, and fatty fish. Most of the discrepancies observed overall were due to the Nutri-Score permitting fewer products for marketing compared with both the WHO-EURO and the NORMA.

### The purpose of the nutrient profiling model and the potential usability of the Nutri-Score

When interpreting the results from the present study, it is important to note that the rationale and purposes behind the development of the WHO-EURO, the NORMA, and the Nutri-Score differ. The WHO-EURO was primarily designed for use by governments to reduce food marketing pressure on children with regard to foods high in energy, saturated fats, trans fatty acids, free sugar, or salt [12]. All 22 food categories are assigned nutrient thresholds, with the principle that “in general, no food should pass or fail the model regardless of its nutrient composition” [12]. However, the categorization process of the WHO-EURO can be complex, as it is not always clear which products belong to which of the 22 categories. This may lead to misclassification of products and there may furthermore be a challenge with low coherence between the criteria for products used in a mixed dish versus when sold as a single product.

The NORMA was also specifically developed to regulate food marketing towards children [13]. It does not cover all food categories, but only those considered most relevant to restrict. The NORMA combines application of nutrient thresholds in five food categories and a complete ban from marketing within six food categories regardless of the nutritional value. Most foods in our dataset (68%) were included in categories with no restrictions. The rationale for banning marketing to children for entire food categories is to avoid the promotion and normalization of consuming foods in these categories, which usually have no or limited nutritional value for children. Including the criteria of no NSS in all five categories with nutrition criteria also precludes reformulation for the sake of avoiding the restrictions. In addition, a limited number of food categories with thresholds simplifies implementation and enforcement. Since 2013, the food industry in Norway has made efforts to avoid marketing of unhealthy foods to children using a model very similar to NORMA through a self-regulatory scheme [34]. This may suggest that this is an acceptable approach also in other European countries wanting to use nutrient profiling models as a basis for similar regulations.

The nutrient profiling model used as a starting point in the development of the Nutri-Score was originally developed by the UK Food Standards Agency to restrict marketing of food products towards children in England [16, 35]. It was further developed and adapted to be used as a FOP nutrition label in France [15], with the current goal of helping consumers identify products with more favourable nutritional values through easier comparison at the point of sale. The Nutri-Score utilizes three algorithms alongside specific rules for cheese and red meat [17, 18], which may make its classification less complex than the WHO-EURO. However, while the data needed to define unhealthy foods according to the WHO-EURO and the NORMA can be found on-pack, there is a need for some additional data to calculate the Nutri-Score, such as the proportion of fruits, vegetables, and legumes in the product. This may represent a challenge if Nutri-Score is not the recommended FOP label in the country and therefore not well-known by the industry.

In October 2022, England implemented new legislation regulating the placement of foods high in fat, sugar, and salt in physical stores (such as aisle ends and checkouts) and their online counterparts [36]. The selected products consisted of pre-packaged items within 13 categories, including sugary soft drinks, crisps, breakfast cereals, confectionery, ice creams, cakes, biscuits, morning goods, desserts, puddings, yoghurt, pizza, potato-based products, and ready meals. Products were identified using the across-the-board nutrient profiling model developed by the UK Food Standards Agency [35]. Evaluations after the implementation revealed support for the legislation from both the retail sector [37] and consumers [38]. Following its introduction, sales on the selected products significantly decreased with an estimated reduction of 220,000 tonnes over a year, equivalent to 2 million fewer items sold per day [39]. However, the nutrient profiling model is not utilized as a FOP label in the UK, presenting some challenges. Similar as with the Nutri-Score, the model requires certain nutritional information that is not typically disclosed on packaging, which complicates matters for both the retail sector and those monitoring the stores’ compliance with the legislation. Therefore, effectively using Nutri-Score to determine which foods cannot be marketed would likely work best if it was a mandatory FOP labelling system. However, under current EU legislation, mandatory front-of-pack nutrition labelling must be implemented at the EU level rather than nationally.

The Nutri-Score assesses the overall nutritional value of foods based on a balance between unfavourable and favourable nutritional components. However, the balancing of components in the Nutri-Score may stimulate manufacturers to manipulate the composition of foods to gain a more favourable rating without improving the actual nutritional value, for example by adding extra fibre, refined carbohydrate, protein, or NSS. Thus, simply using Nutri-Score C-E as criteria may potentially provide loopholes. Our study demonstrated that Nutri-Score would allow marketing of some undesirable products, incoherent with the WHO-EURO and NORMA. Therefore, if the Nutri-Score were to be implemented as a tool to regulate food marketing to protect children, in addition to adding threshold-based criteria, banning marketing within the unhealthiest food categories, similar to NORMA, would limit the risk of unbeneficial product reformulation by food manufacturers.

The Nutri-Score is already in use as a FOP in several European countries, and it may be beneficial if one nutrient profiling model could be used for several purposes. It may be less complex both for the industry, the public, and the authorities to utilize one profile, and it would contribute to coherence in nutrition policies [40–42]. At the same time, we acknowledge that there is ongoing debate regarding whether across‑the‑board nutrient profiling models are preferable to food group‑specific approaches, particularly in regulatory contexts [43, 44]. In this context, some minor, purpose-specific adaptations to the Nutri-Score may be necessary. As argued above, we propose that additional criteria and a complete ban within the unhealthiest food categories, are necessary for the purpose of restricting the marketing of foods to children. It is also important to bear in mind that any nutrient profiling model must be based on the nutritional challenges in the target population and fit to national food cultures. Whilst many nutritional challenges are similar across Europe, there are still variations that may make adaptations to standardized nutrient profiling models necessary.

### The Nutri-Score cut-off

Previous research has explored the agreement between the Nutri-Score 2015 version and the WHO-EURO from 2015 for marketing restriction purposes across a wide range of products in Australia [45]. In the Australian study, Nutri-Score D-E had an overall agreement of 64% with the WHO-EURO. The subcategories cakes, muffins and pastries, fruits, vegetables, chocolates and sweets, and energy drinks demonstrated high levels of agreement. Conversely, the Australian study identified significant disagreement, particularly with products that were permitted by the Nutri-Score 2015 version, but restricted by the WHO-EURO, including breakfast cereals, pre-prepared salads and sandwiches, ready meals, meat alternatives, milk, and yoghurt [45]. In another study conducted in French supermarkets on products specifically marketed for children, the agreement between Nutri-Score (2015) C-E, and the WHO-EURO 2015 was 95% [46]. The Nutri-Score 2015 allowed higher sugar content compared to the updated Nutri-Score from 2023, whereas the sugar thresholds were similar between the WHO-EURO 2015 and WHO-EURO 2023 versions. The updated Nutri-Score likely explains some of the differences in agreement between studies, in addition to variations in cut-off values and food selection. The Nutri-Score cut-off selected to define products for marketing restrictions will largely affect the agreement with the WHO-EURO and the NORMA. Together with previous studies [45, 46], the present study suggests that using Nutri-Score C-E as a cut-off provides a high level of agreement with the WHO-EURO and the NORMA.

### The potential of the “NewTools-score”

The NewTools-score provided results that were overall very similar to those of the Nutri-Score. Better agreement was demonstrated by the NewTools-score within the categories breakfast cereals when compared to the NORMA, and fats when compared to the WHO-EURO. However, it showed poorer agreement within the grains category when compared to the WHO-EURO. This discrepancy arises because the NewTools-score, unlike the Nutri-Score, generally does not assign A or B to refined grains with low fibre content [25]. Since the WHO-EURO does not provide thresholds for fibre content in these food categories, this resulted in a poorer agreement with the NewTools-score. However, overall, this study indicates that the performance of both the Nutri-Score and the NewTools-score in this setting is close to equal. 

### Strengths and limitations

Strengths of the present study include a thorough evaluation of different nutrient profiling models by comparing the Nutri-Score both with the WHO-EURO and NORMA, highlighting its potential implications for marketing restrictions to protect children. Applying the updated versions (2023) of both the WHO-EURO and the Nutri-Score also enhances the relevance of the findings. The results of this study may provide valuable insights for policymakers in selecting appropriate nutrient profiling models for regulating food marketing to children.

However, the study has several limitations. Although we analysed a dataset of 1,944 products, it only included foods represented within the Norwegian food composition table, potentially not capturing the full variety in branded food items in the market. The Norwegian food composition table is designed to capture foods most consumed by the Norwegian population, but it does not allow for sales weighting which may have influenced our results. Furthermore, the study’s emphasis on the Norwegian food composition table may limit the generalizability of the findings to other countries with different dietary habits and regulatory environments.

## Conclusion

This study indicated that the Nutri-Score, and the «NewTools-score», may be used for identifying a high proportion of products unsuitable for marketing to children. However, some additional criteria, including a complete ban from marketing in selected food categories, seems warranted to better protect children from exposure to marketing of unhealthy products. Further, the Nutri-Score requires more data for calculation than can be found on-pack, which may result in some practical challenges if the score is not a mandatory or commonly used FOP.

## Supplementary Information

Below is the link to the electronic supplementary material.

Supplementary file1 (DOCX 2635 kb)

## Data Availability

No datasets were generated or analysed during the current study.
