# Supplementary material for: The potential of Nutri-Score to differentiate foods in regulating food marketing to children
Source: Eur J Nutr. 2026 Jul 30;65(5):222. doi: 10.1007/s00394-026-04041-4 (PMC13424713; doi:10.1007/s00394-026-04041-4)
Supplement: Supplementary file 1 — Supplementary file1 (DOCX 2635 kb) [file 394_2026_4041_MOESM1_ESM.docx]

The Nutri-Score’s potential to differentiate foods in regulating food marketing to children

Anna Amberntsson^a,b†^, Mari Mohn Paulsen^a,b†^*, Jorunn Sofie Randby^c^, Kaja Lund-Iversen^a,b^, Anne Lise Brantsæter^a,b^, Lene Frost Andersen^d^, Marianne Hope Abel^b,c,e^.

^a^Department of Food Safety, Norwegian Institute of Public Health, Oslo, Norway.

^b^Centre for Sustainable Diets, Norwegian Institute of Public Health, Oslo, Norway.

^c^Department of Physical Health and Ageing, Norwegian Institute of Public Health, Oslo, Norway

^d^Department of Nutrition, Institute of Basic Medical Sciences, University of Oslo, Norway.

^e^Centre for Evaluation of Public Health Measures, Norwegian Institute of Public Health, Oslo, Norway.

^†^Shared first authorship

## Overlap between the Nutri-Score and the «NewTools-score»

To provide insight into the similarities between the Nutri-Score and the «NewTools-score», a cross-classification table was created of the products in the Norwegian food composition table (**Supplementary** **Table 1**). The food categories with food items obtaining a different class with the «NewTools-score» were chocolate and confectionary, cakes biscuits, and other sweets, breakfast cereals, cheese, and pasta, rice and grains. Only three products were moved more than one class.

**Supplementary Table 1.** Number of products scored using the Nutri-Score and the «NewTools-score» in the Norwegian food composition table (N=1,944).

| **The Nutri-Score** | **The «NewTools-score»** | | | | | |
| --- | --- | --- | --- | --- | --- | --- |
|  | **A** | **B** | **C** | **D** | **E** | **Total** |
| **A** | 682 | 10 | 1 | 0 | 0 | 693 |
| **B** | 1 | 184 | 29 | 0 | 0 | 214 |
| **C** | 1 | 2 | 357 | 23 | 0 | 383 |
| **D** | 0 | 1 | 9 | 274 | 84 | 368 |
| **E** | 0 | 0 | 1 | 5 | 280 | 286 |
| **Total** | 684 | 197 | 397 | 302 | 364 | 1,944 |

**Supplementary Table 2.** Foods and beverages in the Norwegian food composition table within each category of the WHO nutrient profile model 2023 (WHO-EURO) and their «NewTools-score» (N=1,944).

| **WHO Category number** | **Food category** | **Categorization by the WHO-EURO** | **«NewTools-score» N (%)^1^** | | | | |
| --- | --- | --- | --- | --- | --- | --- | --- |
|  |  |  | **A** | **B** | **C** | **D** | **E** |
|  | **All foods** | **Not permitted (n=919)**  **Permitted (n=1,025)** | **37 (4)**  **647 (63)** | **46 (5)**  **151 (15)** | **208 (23)**  **189 (18)** | **272 (30)**  **30 (3)** | **356 (39)**  **8 (1)** |
| 1 | Chocolate and sugar confectionery, energy bars, sweet toppings and desserts | Not permitted (n=87)  Permitted (n=7) | 2 (2)  5 (71) | 2 (2)  0 (0) | 9 (10)  0 (0) | 10 (11)  0 (0) | 64 (74)  2 (29) |
| 2 | Cakes, sweet biscuits and pastries; other sweet bakery wares | Not permitted (n=179)  Permitted (n=0) | 2 (1) 0 (0) | 4 (2) 0 (0) | 26 (15) 0 (0) | 29 (16) 0 (0) | 118 (66) 0 (0) |
| 3 | Savoury snacks | Not permitted (n=36)  Permitted (n=23) | 0 (0) 18 (78) | 4 (11)  3 (13) | 14 (39)  2 (9) | 9 (25)  0 (0) | 9 (25)  0 (0) |
| 4.1 | Juices | Not permitted (n=30)  Permitted (n=0) | 0 (0) 0 (0) | 3 (10) 0 (0) | 12 (40) 0 (0) | 10 (33) 0 (0) | 5 (17) 0 (0) |
| 4.2 | Dairy milk drinks | Not permitted (n=28)  Permitted (n=28) | 0 (0)  0 (0) | 3 (11)  25 (89) | 11 (39)  3 (11) | 3 (11)  0 (0) | 11 (39)  0 (0) |
| 4.3 | Plant based milk | Not permitted (n=12)  Permitted (n=15) | 0 (0)  0 (0) | 7 (58)  2 (13) | 1 (8)  7 (47) | 0 (0)  6 (40) | 4 (3)  0 (0) |
| 4.4 | Energy drinks | Not permitted (n=2) Permitted (n=0) | 0 (0) 0 (0) | 0 (0) 0 (0) | 1 (50) 0 (0) | 0 (0) 0 (0) | 1 (50) 0 (0) |
| 4.5 | Soft drinks, bottled waters and other drinks | Not permitted (n=22) Permitted (n=16) | 0 (0)  7 (44) | 0 (0)  6 (38) | 5 (23)  2 (12) | 3 (14)  1 (6) | 14 (64)  0 (0) |
| 5 | Edible ices | Not permitted (n=30) Permitted (n=0) | 3 (10) 0 (0) | 0 (0) 0 (0) | 2 (7) 0 (0) | 16 (53) 0 (0) | 9 (30) 0 (0) |
| 6 | Breakfast cereals | Not permitted (n=13) Permitted (n=12) | 0 (0)  5 (42) | 1 (8)  1 (8) | 4 (31)  5 (42) | 6 (46)  1 (8) | 2 (15)  0 (0) |
| 7 | Yoghurt, sour milk, cream and similar foods | Not permitted (n=33) Permitted (n=15) | 2 (6)  6 (40) | 8 (24)  4 (27) | 14 (42)  4 (27) | 4 (12)  1 (6) | 5 (15)  0 (0) |
| 8 | Cheese | Not permitted (n=56)  Permitted (n=13) | 0 (0)  1 (8) | 0 (0)  0 (0) | 5 (9)  4 (31) | 23 (41)  3 (23) | 28 (50)  5 (38) |
| 9 | Ready-made and convenience foods and composite dishes | Not permitted (n=48)  Permitted (n=102) | 0 (0)  24 (24) | 0 (0)  31 (30) | 27 (56)  42 (41) | 19 (40)  5 (5) | 2 (4)  0 (0) |
| 10 | Butter, other fats and oils | Not permitted (n=21)  Permitted (n=31) | 0 (0)  0 (0) | 0 (0)  12 (39) | 0 (0)  16 (52) | 5 (24)  2 (6) | 16 (76)  1 (3) |
| 11 | Bread, bread products and crisp breads | Not permitted (n=20)  Permitted (n=90) | 1 (5)  42 (47) | 0 (0)  15 (17) | 7 (35)  32 (36) | 8 (40)  1 (1) | 4 (20)  0 (0) |
| 12 | Fresh or dried pasta, rice and grains | Not permitted (n=3)  Permitted (n=103) | 0 (0)  57 (55) | 0 (0)  17 (17) | 1 (33)  28 (27) | 2 (67)  1 (1) | 0 (0)  0 (0) |
| 13 | Fresh and frozen meat, poultry, fish and similar | Not permitted (n=12)  Permitted (n=126) | 0 (0)  108 (86) | 3 (25)  5 (4) | 1 (8)  11 (9) | 7 (58)  2 (2) | 1 (8)  0 (0) |
| 14 | Processed meat, poultry, fish and similar | Not permitted (n=165)  Permitted (n=159) | 4 (2)  105 (66) | 5 (3)  19 (12) | 24 (15)  29 (18) | 87 (53)  6 (4) | 45 (27)  0 (0) |
| 15 | Fresh and frozen fruit, vegetables and legumes | Not permitted (n=0)  Permitted (n=215) | 0 (0)  212 (98) | 0 (0)  2 (1) | 0 (0)  0 (0) | 0 (0)  1(1) | 0 (0)  0 (0) |
| 16 | Processed fruit and vegetables | Not permitted (n=59)  Permitted (n=51) | 13 (22)  45 (88) | 3 (5)  6 (12) | 27 (46)  0 (0) | 12 (20)  0 (0) | 4 (7)  0 (0) |
| 17 | Savoury plant-based foods/meat analogues | Not permitted (n=28)  Permitted (n=11) | 7 (25)  9 (82) | 2 (7)  1 (9) | 7 (25)  1 (9) | 6 (21)  0 (0) | 6 (21)  0 (0) |
| 18 | Sauces, dips and dressings | Not permitted (n=35)  Permitted (n=8) | 3 (9)  3 (38) | 1 (3)  2 (25) | 10 (29)  3 (38) | 13 (37)  0 (0) | 8 (23)  0 (0) |

^1^Percentages may not sum to 100% due to rounding

**Supplementary Table 3.** Foods and beverages in the Norwegian food composition table within each category of the nutrient profiling model from the Norwegian Regulation prohibiting the marketing of certain foods particularly aimed at children (NORMA) and their «NewTools-score» (N=1,944).

| **Category number** | **Food category** | **Categorization by the NORMA** | **«NewTools-score»  N (%)^1^** | | | | |
| --- | --- | --- | --- | --- | --- | --- | --- |
|  |  |  | **A** | **B** | **C** | **D** | **E** |
|  | **All foods** | **Not permitted (n=452)**  **Permitted (n=1,492)** | **7 (2) 671 (45)** | **24 (5) 177 (12)** | **99 (22) 297 (20)** | **91 (20) 214 (14)** | **231 (51) 133 (9)** |
| 1 | Chocolate and confectionary, energy bars, sweet spreads, and desserts | Not permitted (n=116)  Permitted (n=0) | 3 (3)  0 (0) | 4 (4)  0 (0) | 14 (13)  0 (0) | 16 (14)  0 (0) | 74 (67)  0 (0) |
| 2 | Cakes biscuits, and other sweet and/or fatty pastries | Not permitted (n=140)  Permitted (n=0) | 0 (0)  0 (0) | 2 (2)  0 (0) | 11 (8)  0 (0) | 18 (13)  0 (0) | 108 (77)  0 (0) |
| 3 | Snacks | Not permitted (n=34)  Permitted (n=0) | 0 (0)  0 (0) | 4 (12)  0 (0) | 12 (35)  0 (0) | 11 (32)  0 (0) | 7 (21)  0 (0) |
| 4 | Ice cream | Not permitted (n=30)  Permitted (n=0) | 3 (10)  0 (0) | 0 (0)  0 (0) | 2 (7)  0 (0) | 16 (53)  0 (0) | 9 (30)  0 (0) |
| 5 | Energy drinks | Not permitted (n=2)  Permitted (n=0) | 0 (0)  0 (0) | 0 (0)  0 (0) | 1 (50)  0 (0) | 0 (0)  0 (0) | 1 (50)  0 (0) |
| 6 | Soft drinks, cordials, and similar drinks | Not permitted (n=21)  permitted (n=0) | 0 (0)  0 (0) | 0 (0)  0 (0) | 6 (29)  0 (0) | 2 (10)  0 (0) | 13 (62)  0 (0) |
| 7 | Juice and similar products | Not permitted (n=3) Permitted (n=27) | 0 (0)  0 (0) | 0 (0)  2 (7) | 2 (67)  10 (37) | 0 (0)  10 (37) | 1 (33)  5 (19) |
| 8 | Milk and plant-based drinks | Not permitted (n=35) Permitted (n=55) | 0 (0)  0 (0) | 9 (26)  33 (60) | 11 (31)  16 (29) | 3 (9)  6 (11) | 12 (34)  0 (0) |
| 9 | Breakfast cereals | Not permitted (n=19) Permitted (n=9) | 1 (5)  6 (67) | 1 (5)  1 (11) | 8 (42)  2 (22) | 7 (37)  0 (0) | 2 (11)  0 (0) |
| 10 | Yoghurt and similar products | Not permitted (n=12) Permitted (n=6) | 3 (25)  3 (50) | 3 (25)  2 (33) | 6 (50)  1 (17) | 0 (0)  0 (0) | 0 (0)  0 (0) |
| 11 | Ready meals and composite dishes | Not permitted (n=49) Permitted (n=61) | 0 (0)  18 (30) | 1 (2)  21 (34) | 27 (55)  22 (36) | 17 (35)  0 (0) | 4 (8)  0 (0) |
| 12 | Other foods | All permitted (n=1,331) | 647 (49) | 114 (9) | 245 (18) | 197 (15) | 128 (10) |

^1^Percentages may not sum to 100% due to rounding

When the «NewTools-score» was applied instead of the Nutri-Score, the overall agreement with the WHO-EURO was 84.5% (**Supplementary Figure 1**). The difference in agreement (n=5) between the second scenario of the Nutri-Score and the «NewTools-score» with the WHO-EURO was because of the «NewTools-score» classifying refined bread, pasta and rice as C and sunflower oil and sesame oil as B, while Nutri-Score classifies them as B and C respectively.

**Supplementary Figure 1**. Overall and within food category agreement between the WHO-EURO and the two scenarios of how the «NewTools-score» could be applied to regulate which food items could be permitted to be marketed to children, in the Norwegian food composition table (N=1,944).

Nutri-Score scenario 1 (S1) – Products are not permitted for marketing if they have «NewTools-score» C-E.


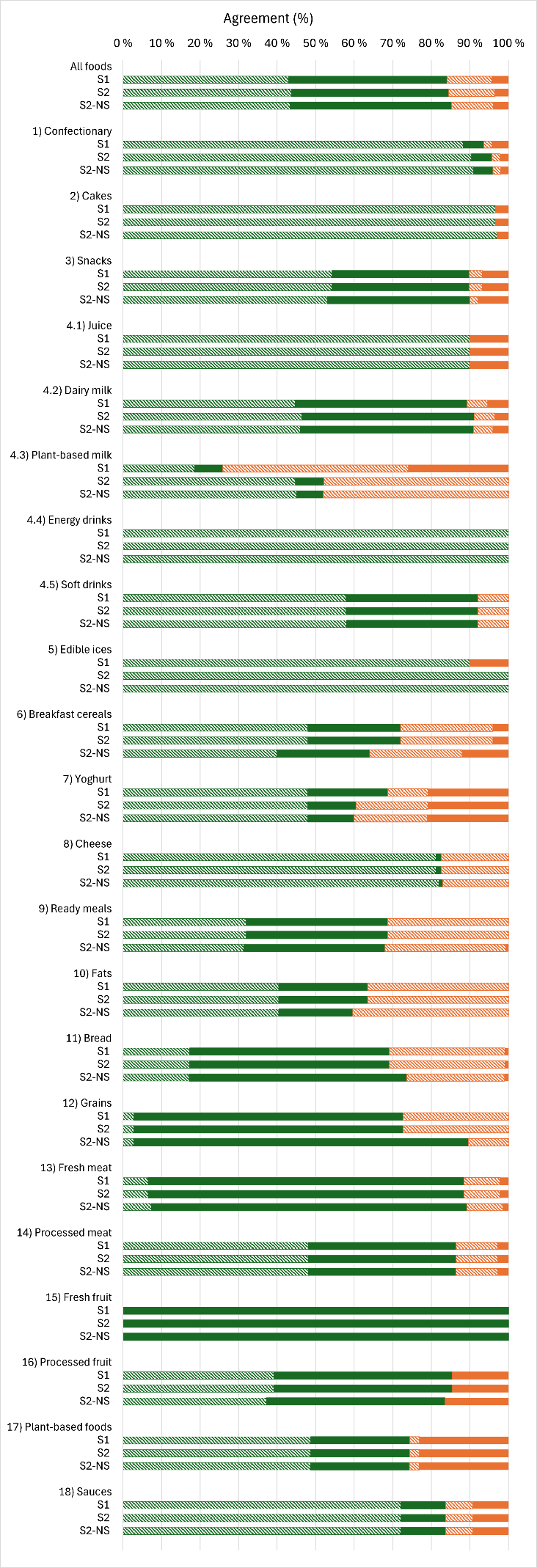

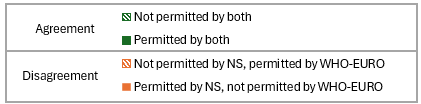


Nutri-Score scenario 2 (S2) – Products are not permitted for marketing if they have «NewTools-score» C-E, contain ≥1 gram of industrially produced trans fatty acids in solid foods, include non-sugar sweeteners in solid foods and beverages, or have added sugar in beverages.

Nutri-Score scenario S2-NS – Same as scenario 2 but with the Nutri-Score instead of the «NewTools-score».
Abbreviations: NS, Nutri-Score; WHO-EURO, the WHO nutrient profile model 2023.

When the «NewTools-score» was applied instead of the Nutri-Score, the overall agreement with the NORMA was 96% (**Supplementary Figure 2**). The difference in agreement (n=3) between the Nutri-Score and the «NewTools-score» with the NORMA was due to the «NewTools-score» classifying two breakfast cereals (13-17% sugar) and puffed rice as C, while Nutri-Score classifies them as B.

**Supplementary Figure 2**. Overall and within food category agreement between the NORMA and the two scenarios of how the «NewTools-score» could be applied to regulate which food items could be permitted to be marketed to children, in the Norwegian food composition table (N=1,944).
Nutri-Score scenario 1 (S1) – Products are not permitted for marketing if they have «NewTools-score» C-E.


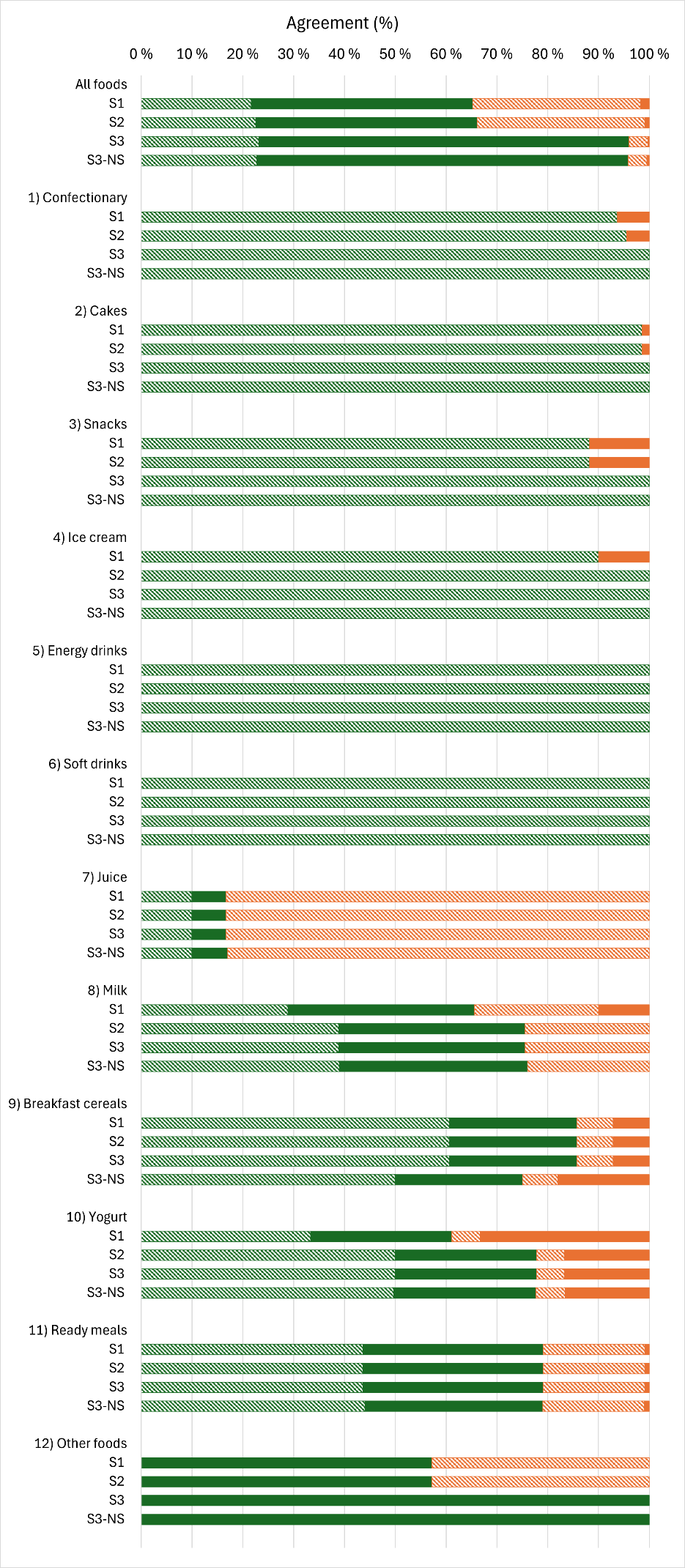

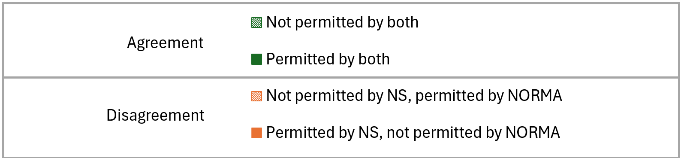


Nutri-Score scenario 2 (S2) – Products are not permitted for marketing if they have «NewTools-score» C-E, contain ≥1 gram of industrially produced trans fatty acids in solid foods, include non-sugar sweeteners in solid foods and beverages, or have added sugar in beverages.

Nutri-Score scenario 3 (S3) – Products are not permitted for marketing if they have «NewTools-score» C-E, contain ≥1 gram of industrially produced trans fatty acids in solid foods, include non-sugar sweeteners in solid foods and beverages, or have added sugar in beverages. In addition, no products in category 1-6 were permitted to be marketed, but all products in category 12 were.
Scenario S3-NS – Same as scenario 3 but with the Nutri-Score instead of the «NewTools-score».
Abbreviations: NS, Nutri-Score; NORMA, NORMA, the nutrient profiling model from the Norwegian Regulation prohibiting the marketing of certain foods particularly aimed at children.
